# USE OF PATIENT PORTALS FOR MEDICATION-RELATED COMMUNICATION: IMPLICATIONS FOR DEPRESCRIBING INTERVENTIONS

**DOI:** 10.1093/geroni/igae098.0662

**Published:** 2024-12-31

**Authors:** Ariel Green, Aleksandra Wek, Kelly Gleason, Mingche Wu, Mary Jo Gamper, Jennifer Wolff

**Affiliations:** Johns Hopkins University School of Medicine, Baltimore, Maryland, United States; Johns Hopkins University, Baltimore, Maryland, United States; Johns Hopkins University, Baltimore, Maryland, United States; Johns Hopkins University, Baltimore, Maryland, United States; Johns Hopkins University, Baltimore, Maryland, United States; Johns Hopkins University, Baltimore, Maryland, United States

## Abstract

People living with dementia (PLWD) often use potentially inappropriate medications (PIM), in which the risks of medication use outweigh the benefits. Little is known about how PLWD and their care partners use patient portals in general, and specifically to communicate with healthcare providers about medications. Our objective was to characterize the content of patient portal messages relating to medications among PLWD, care partners, and clinicians in a single health system, to inform the development of a portal-based intervention to reduce the use of PIM. We examined secure message exchanges between 2/18/2017 and 10/2/2022 from patients 65 years and older with dementia or their care partners in an integrated, academic health system. A total of 399 message threads from 159 unique patients were analyzed. Patients were on average 78.4 years old (SD 8.0). The majority (65%) were female, white (76%), and non-Hispanic/Latinx (96%); 15% had at least one registered proxy portal user. The most common topic raised was logistics (42%), such as clarification of medication instructions. This was followed by messages about adverse effects/treatment burden (25%), asking for new medications (23%), and openness to stopping a medication (21%). Findings suggest that the patient portal is frequently used by PLWD and their care partners to discuss medication-related concerns and that it is a promising platform for delivering interventions to reduce the use of PIM in this population.

